# Improving testing capacity for COVID-19: experiences and lessons from Senegal, Uganda, Nigeria, and the Democratic Republic of Congo

**DOI:** 10.3389/fpubh.2023.1202966

**Published:** 2023-11-16

**Authors:** Marc Bosonkie, Landry Egbende, Alice Namale, Olufunmilayo I. Fawole, Ibrahima Seck, Susan Kizito, Didine Kaba, Suzanne N. Kiwanuka, Issakha Diallo, Segun Bello, Steven N. Kabwama, Yves Kashiya, Fred Monje, M. D. Dairo, Berthold Bondo, Noel Namuhani, Mamadou M. M. Leye, A. S. Adebowale, Oumar Bassoum, Eniola A. Bamgboye, Manel Fall, Mobolaji Salawu, Rotimi Afolabi, Rawlance Ndejjo, Rhoda K. Wanyenze, Mala Ali Mapatano

**Affiliations:** ^1^Department of Nutrition, Kinshasa School of Public Health, Faculty of Medicine, University of Kinshasa, Kinshasa, Democratic Republic of Congo; ^2^Department of Disease Control and Environmental Health, School of Public Health, Makerere University, Kampala, Uganda; ^3^Faculty of Public Health, College of Medicine, University of Ibadan, Oyo, Nigeria; ^4^Department of Preventive Medicine and Public Health, Cheikh Anta Diop University, Dakar, Senegal; ^5^Department of Biostatistics and Epidemiology, Kinshasa School of Public Health, Faculty of Medicine, University of Kinshasa, Kinshasa, Democratic Republic of Congo; ^6^Department of Health Policy, Planning and Management, School of Public Health, College of Health Sciences, Makerere University, Kampala, Uganda; ^7^Barumbu General Hospital, Kinshasa, Democratic Republic of Congo; ^8^Department of Health Policy Planning and Management, School of Public Health, Makerere University, Kampala, Uganda

**Keywords:** COVID-19 testing capacity, experiences, lessons, COVID-19 response, Africa

## Abstract

**Background:**

African countries leveraged testing capacities to enhance public health action in response to the COVID-19 pandemic. This paper describes experiences and lessons learned during the improvement of testing capacity throughout the COVID-19 response in Senegal, Uganda, Nigeria, and the Democratic Republic of the Congo (DRC).

**Methods:**

The four countries’ testing strategies were studied using a mixed-methods approach. Desk research on COVID-19 testing strategies was conducted and complemented by interviewing key informants. The findings were synthesized to demonstrate learning outcomes across the four countries.

**Results:**

The four countries demonstrated severely limited testing capacities at the onset of the pandemic. These countries decentralized COVID-19 testing services by leveraging preexisting laboratory systems such as PCR and GeneXpert used for the diagnosis of tuberculosis (TB) to address this gap and the related inequities, engaging the private sector, establishing new laboratories, and using rapid diagnostic tests (RDTs) to expand testing capacity and reduce the turnaround time (TAT). The use of digital platforms improved the TAT. Testing supplies were sourced through partners, although access to global markets was challenging. Case detection remains suboptimal due to high costs, restrictive testing strategies, testing access challenges, and misinformation, which hinder the demand for testing. The TAT for PCR remained a challenge, while RDT use was underreported, although Senegal manufactured RDTs locally. Key findings indicate that regionally coordinated procurement and manufacturing mechanisms are required, that testing modalities must be simplified for improved access, and that the risk-based testing strategy limits comprehensive understanding of the disease burden.

**Conclusion:**

Although testing capacities improved significantly during the pandemic, case detection and access to testing remained suboptimal. The four countries could benefit from further simplification of testing modalities and cost reduction. Local manufacturing and pooled procurement mechanisms for diagnostics are needed for optimal pandemic preparedness and response.

## 1. Introduction

The coronavirus disease 2019 (COVID-19) pandemic is an unprecedented global health crisis that has affected almost all countries and territories worldwide, causing ~6.5 million deaths among 600 million cases globally.^1^

Countries and continents have grappled with the unpredictable and dramatic consequences of a virus that spread rapidly among populations. Even in better-prepared health settings, testing capacities and public health reporting systems were challenged by the rapid local transmission of severe acute respiratory syndrome coronavirus 2 (SARS-CoV-2) (1). For example, England’s decision to stop community testing and contact tracing for COVID-19 in early March 2020 was partly driven by a lack of testing capacity. On March 12, 2020, as cases of the virus soared, the government announced that it would stop all community testing for COVID-19 and focus instead on testing people in hospitals and protecting health workers (2).

In Africa, to improve testing capacity, the African Development Bank provided $2 million in emergency funds to help the World Health Organization (WHO) strengthen its capacity to support African countries (3). The United Nations Office for the Coordination of Humanitarian Affairs (OCHA) reported that as of June 2020, countries rehabilitated their unsuitable laboratories or built laboratories where none existed. By the end of November 2020, almost all African countries were able to test their populations. Some countries even had fairly efficient laboratories capable of performing viral genetic sequencing (3).

The WHO emphasized the importance of testing during COVID-19 (4) and for these countries to develop national laboratory capacity (5); however, actual testing capacity is often not analyzed when deploying national diagnostic strategies. Despite these interventions, the testing capacity for COVID-19 remained low due to the large population size compared to the availability of the tests in many Sub-Saharan African (SSA) countries. Consequently, the reported number in low-income countries may not reflect the actual burden of the pandemic (6).

Countries in SSA employed various strategies to monitor, identify, and improve testing strategies, providing an opportunity for cross-country analysis and synthesis of promising practices for adaptation. This paper describes experiences and lessons learned in the improvement of testing capacities during the COVID-19 response in Senegal, Uganda, Nigeria and the Democratic Republic of Congo (DRC) to provide information regarding the response to COVID-19 and for future pandemics in the region and globally.

## 2. Materials and methods

### 2.1. Study area

A multicountry study was conducted in Nigeria, Senegal, Uganda, and DRC to assess the testing capacity in the four countries. The DRC is located in Central Africa, Nigeria and Senegal in West Africa, and Uganda in East Africa. All four countries have experienced public health emergencies of international concern, particularly filo virus disease outbreaks, including Ebola and Marburg virus disease outbreaks. Senegal and Nigeria registered cases during the 2014–2016 Ebola virus disease outbreak that affected West Africa, while Uganda and the DRC have reported several Ebola virus disease outbreaks over the past 2 decades (7).

### 2.2. COVID-19 in the study countries

All the four countries reported their first cases of COVID-19 between February and March 2020. Nigeria confirmed its first case on February 27, Senegal on March 2, the DRC on March 10, and Uganda on March 20. In the first 6 months of the pandemic between February and August 2020, Uganda had the lowest daily new confirmed cases per million people, followed by the DRC and Nigeria with Senegal having the highest rates. Between August and December 2020, however, Uganda had the highest daily new confirmed cases per million people, followed by Senegal, Nigeria, and the DRC. As of March 2021, the four countries experienced at least three waves of the pandemic.

### 2.3. Study design and data collection

This was a descriptive qualitative study conducted in two phases, a desk review and in-depth interviews of key informants (KI) in the four countries. This paper focuses on testing capacities. Data were collected across the four countries from November 2020 to March 2021. Key informants were selected by purposive sampling and included policy-makers and laboratory managers in each level of the health system. The interviews were conducted either over the phone, using internet electronic communication platforms like Zoom™ or in-person with strict observance of COVID-19 standard prevention operating procedures like physical distancing and mask wearing. Interviewers visited the offices of the informants at their convenient time to conduct the KI interviews. A total of 110 KIs were included, 22 in DRC, 32 in Nigeria, 21 in Senegal, and 35 in Uganda. All were interviewed on the following themes: country’s previous experience with outbreak responses, country’s previous experience with testing, COVID-19 testing experiences, sourcing COVID-19 testing supplies, expansion of testing capacity, improving access to testing. Two data collection instruments were used. The first instrument was a literature abstraction form (Additional file 1) used to gather information across the four countries on testing modalities and changes in testing criteria. The data extraction form was developed and piloted in the excel format but was also applied in the word document format to extract data, depending on preference by country teams. The second instrument was a KI interview guide (Additional file 2), which was used to obtain information on the strengths and weaknesses of the testing in the four countries. In each country, data collection was facilitated by skilled research assistants with proficiency in qualitative research. The research assistants received additional training on data collection strategies and use of the guide. Prior to conducting the KIIs, the research team conducted a desk research. The mixed-methods approach was utilized to facilitate the triangulation of information. Key learnings both similar and unique to a specific country context were synthesized across countries.

### 2.4. Desk review

The desk review focused on various government resources, including but not limited to preparedness and response plans; protocols, such as standard operating procedures (SOPs) and guidelines for diagnosis and testing; daily and monthly COVID-19 reports; minutes of COVID-19 intervention meetings; and data from websites including government COVID-19 platforms, local non-governmental and international organization websites such as WHO, UNICEF, and USAID among others. These resources were collected from the departments of the Ministry of Health and downloaded from the internet. The keywords used for the internet search included COVID-19 OR Corona virus OR Corona virus disease AND testing OR screening OR PCR OR GeneXpert OR Rapid diagnostic test limited to the four countries of the study. Those articles or documents that were deemed to contain elaborate information on COVID-19 testing were included. The research focused on (i) the elements of the testing value chain, from the clinical ordering of a test to the return of test results; (ii) key challenges and strengths in testing; (iii) the country’s previous experience with testing; (iv) the country’s previous experience with outbreak responses; and (v) the results of indicators such as the number of tests conducted, confirmed cases, contacts traced, and COVID-19-related deaths.

### 2.5. Key informant interviews

Key informants were interviewed to build on the literature review and further explore aspects of COVID-19 testing. The interview guide incorporated several themes, including coordination, surveillance and contact tracing, laboratory testing, case management and continuity of essential services. Findings related to laboratory testing experiences were the focus of this paper. The interviews were conducted face-to-face (observing COVID-19 protective measures), by phone, or through communication platforms such as Zoom™ or Teams.

### 2.6. Data management and analysis

The desk review findings were synthesized using manually generated themes. All interviews were audio-recorded and transcribed verbatim. The themes were manually synthesized into subthemes and themes. The results are presented with quotes to supplement and reinforce the findings from the desk review and key informant interviews.

### 2.7. Ethical considerations

Each country obtained ethical approval to conduct the study procedures as part of the COVID-19 Assessment Project. In the Democratic Republic of Congo, the study was approved by the Kinshasa School of Public Health Ethics Committee with the number ESP/CE/198/2020 on December 2, 2020; in Nigeria, the study was approved by the National Health Research Ethics Committee, Abuja, with the number NHREC/01/01/2007; in Senegal, the University of Dakar School of Public Health Ethics Committee approved the study with the number 00030/MSAS/CNERS/SP on March 2, 2021; and in Uganda, the study was approved by the Makerere University School of Public Health Higher Degrees Research for Uganda with the number IRB00011353. The initial tools were in English and translated into French for application in the DRC and Senegal.

## 3. Results

110 results were included, 22 for the DRC, 32 for Nigeria, 21 for Senegal, and 35 for Uganda. For each county, half interviews were conducted with policy-makers and half with laboratory managers. All were interviewed on the following themes: country’s previous experience with outbreak responses, country’s previous experience with testing, COVID-19 testing experiences, sourcing COVID-19 testing supplies, expansion of testing capacity, and improving access to testing.

### 3.1. COVID-19 testing experiences

By January 2, 2021, DRC had conducted 95,443 cumulative COVID-19 tests, representing a test *per capita* of 1.2/1,000, Nigeria had conducted 952,975 COVID-19 tests (test *per capita* of 4.6/1,000), Uganda 753,602 COVID-19 tests (test *per capita* of 16.2/1,000), and Senegal 16.657 tests per 1,000.^2^

### 3.2. Sourcing COVID-19 testing supplies

It was estimated that as of December 2020, up to 80% of the testing reagents and supplies in the DRC had been donated by partners, including the WHO, US-CDC, USAID, UNICEF, the British Cooperation, China, Japan, Médecin Sans Frontière (MSF), the Global Fund, and the Government of Israel. The remaining 20% was acquired by the government through a World Bank-funded “Projet de Développement du Système de Santé” project (8).^3^

*“Initially, when the pandemic started, the partners had not yet produced any results*. *We had to resort to the existing stock of tests to perform the tests*. *As a public health laboratory, we had to resort to the stock of tests intended for the diagnosis of influenza that the School of Public Health of Kinshasa provides us with once a year*. *We had no choice*. *And when this stock ran out, while waiting for the partners to react, as it is not a matter of “tick to tock” with the partners, you have to place the order, it has to be evaluated, and so on… by the time it arrives, it takes all the time… During the first wave, we had to use this stock of flu tests*. *When it ran out, we had to resort to the stocks of the side laboratories”* (*KI1*, *Institut National de Recherche Biomédicale, Kinshasa, DRC*).

*“What existed was that through the cooperation or partnership with WHO, which essentially supported the influenza surveillance laboratory, we received all the inputs to carry out the test,* i.e.*, primers, transport media, culture media,* etc. *Today, we still receive the tests through this partnership*. *At the INRB level, as we also use the COVID voyage tests and thanks to our Indian partner, we do the supply by ourselves by purchasing the COVID voyage tests”* (*KI3, Program National de Lutte contre la Tuberculose, Kinshasa*).

Prior to the COVID-19 outbreak, Nigeria conducted a quantification exercise using projected numbers to estimate the need for testing supplies (including PPE). The Federal Government of Nigeria immediately released 5 billion naira (US$12.5 million) in special intervention funds and later an additional 10 billion naira (US$25 million) to the Lagos state government, as Lagos was at the epicenter of the outbreak (9). This funding included support for the procurement of testing supplies. Additional funding for testing supplies was made available through donor partnerships. Nigeria did manufacture RDTs locally, although several kits were in the developmental stage at the National Institute of Medical Researchers (NIMR), Lagos.

*“Sourcing the testing supplies, so for the RDTs we have some that are been sourced by procured at the National level and now distributed to State, we also have some that are being supplied by partners”* (*KI-1, Nigeria Centers for Disease Control and Prevention* (*NCDC*)*, Abuja, Nigeria*).

*“At the initial stage, testing supply were sourced from the national, although some were produced by the laboratories at different levels*. *Gradually the state produced some radiant while NCDC also produced some*. *At different levels the testing supplies has been sourced from state procurement and NCDC”* (*KI-3, MoH, Nigeria*).

In Senegal, at the onset of the COVID-19 outbreak, testing reagents and supplies were donated to the government by partners, especially the Clinton Health Access Initiative (CHAI), the Global Fund (GF), and the WHO. The procurement of the COVID-19 testing reagents and supplies was managed by the Government of Senegal through the Directorate of Pharmacy and Medicine (Drug Regulatory Authority). The procurement process followed the supply logistics scheme of the National Pharmacy Supply. Senegal began manufacturing antigen RDTs in July 2020 through the DiaTropix project, which was formally launched in November 2020 to support diagnostics.^4^^,^^5^ The test kit was estimated to cost $1 and provide results in 10 min. The RDTs were piloted in four of 14 regions (July 2020–January 2021), and countrywide roll out began in January 2021. The project donated up to 70,000 RDTs to the government. Production capacity was expected to reach 4 million units annually, with plans to export the kits regionally.^6^

*“There are several levels, there are the reagents that are donated and that the government receives but also the laboratories are supplied within the framework especially with the travelers who had to do their tests and it was the responsibility of the lab*. *So the labs were buying tests outside of what the government was donating”* (*KI1, Institute Pasteur, Dakar, Senegal*).

*“At one point, there was a stock tension and the Ministry at the central level sent an email to ask for better rationalization of the tests because there was a shortage that was coming if we were not careful*. *It was the moment when we had an exponential increase of cases at the end of December and the central level had told us that they had ordered cartridges at the international level and that there was a stock shortage*. *So, they could not get their order while what was left here was running out”* (*KI7, Health Center Medical Officer, Senegal*).

In Uganda, the government, through the Ministry of Health, procured testing supplies from other countries worldwide; for instance, PPE was obtained from China, while PCR amplification reagents were obtained from Germany and the United States of America. The initial testing supplies were mainly sourced as donations or procured through funding from partners, such as the WHO, the United Kingdom, the Danish Government, the World Bank (approximately 90%), the private sector, and the government.^7^ Quantification of the required supplies conducted during preparedness evaluations for COVID-19 testing helped establish mechanisms to address gaps. Strategies to improve the procurement processes included pooled procurement through Global Fund support and the use of multiple suppliers, such as China, the United States, and Germany. Uganda has yet to manufacture testing supplies; however, the country is developing RDTs. An antibody RDT was launched on March 18, 2021, by Makerere University. The rapid antibody test kit was expected to cost approximately one dollar and provide results in 2–5 min. The development of this kit was supported by the public and private sectors, including the Government of Uganda, Makerere University through the Research and Innovations Fund, the French Embassy in Uganda, the Uganda Bankers Association, and Astel Diagnostics Uganda, a WHO certified manufacturer.^8^ The kit underwent field validation before being released to the market in 2022.

### 3.3. Expansion of testing capacity

At the start of the epidemic in the DRC, only the National Reference Public Health Laboratory (INRB) performed COVID-19 testing, with a capacity of 200 tests per day (March to June 2020). As of December 2020, the daily testing capacity had increased to approximately 2,000 PCR tests per day. Testing services were decentralized in June 2020, and the number of testing laboratories increased to 25 in 14 of the 26 provinces, covering an estimated 60% of the population by December 2020.

The expansion of COVID-19 testing sites was guided by the availability of infrastructure (human resources and GeneXpert equipment), disease epidemiologists, laboratories in Kinshasa, (the epicenter), and considerations of geographical equity. The GeneXpert equipment was previously used for TB diagnostics, and each of the 22 laboratories performed 20–30 tests a day. Antibody RDTs were integrated into the testing algorithm in June 2020 to further increase testing capacity. Additional capacity building was achieved through training at least 255 healthcare workers and the dissemination of SOPs. Training content focused on sample collection, storage, and diagnostics.

The number of tests performed increased monthly from March to December 2020 as the testing capacity improved. By the end of December 2020, a high-level PCR lab was set up at the University of Kinshasa to increase capacity further.

*“I have written a circular note to all my coordinating doctors to let GeneXpert be used concomitantly not only for COVID but also for HIV, monkeypox, Ebola, or any other pathology in a vision of strengthening the health system*. *This is how the PNLT has contributed to the extension of the diagnosis of COVID and this is an experience for which the DRC should be very proud and capitalize if there are best practices, this kind of best practices must be made known to people*. *We had also specified that for the best use, the same technician who does the TB test should do the COVID because he is the one who knows the machine and knows how to handle it, he will organize his time by saying to himself, for example, that in the morning he will do everything that is TB and in the afternoon, the COVID”* (*KI3, Program National de Lutte contre la Tuberculose, Kinshasa, DRC*).

*“We had to obtain the support of partners who provided us with inputs such as the CDC, the African Union who had really supported African countries with reagents and consumables*. *There are also the traditional partners of INRB such as WHO which is always with us, JICA, CHAI, MSF* (*which also helps us with consumables*)*, UNICEF, the Global Fund which has also just been activated with the first order made since the first wave…as I told you earlier with the partners it is not from tick to tock…it is now that they are delivering everything that we ordered during the first wave*. *This is what allows us to improve our testing capacity”* (*KI1, Head of the respiratory virus laboratory in the Department of Virology, Institut National de Recherche Biomédicale, Kinshasa, DRC*).

In March 2020, Nigeria had three laboratories and could conduct 1,500 tests daily. By October 2020, there were 69 testing laboratories with a total daily output of 15,000–20,000 tests (excluding private labs).^9^ The distribution of testing laboratories was guided by case load, population density, and equity, with priority given to the epicenters (Lagos, Abuja, and Kano), including geopolitical zonal distribution. Of note, 45 of the 69 public labs use open PCR, while others use different platforms, including GeneXpert, Abbott, and Cobas.

*“So the testing capacity, as the cases kept increasing, there was a need to actually get the true picture of the prevalence or the numbers of cases in the country; so there was a need to increase the testing capacity across the state; so that led to the establishment of labs in the state contributing to the improvement of the number of samples being collected*. *Another thing that was done was to establish sample collection sites*. *Before then, sample collections were kind of being centralized; people will had to travel down to some places to have their samples collected*. *But after a while it was decentralized in such a way that there was establishment of sample collecting sites at least one per Local Government Area, so this enabled people at the lower level to have their samples collected promptly”* (*KI-1, NCDC, Abuja, Nigeria*).

*“At the early stage of the pandemic, samples were taken from different centers to Abuja for test, but over time laboratories were decentralized*. *For example, Oyo State has one laboratory, but as the pandemic moved on, the State is working on having the second laboratory to ensure fast release of COVID-19 results”* (*KI-3,MoH, Nigeria*).

In Senegal, the testing capacity improved rapidly between March 2020 and July 2020: the number of laboratories capable of testing for COVID-19 and the number of tests that could be performed by the laboratories increased. The number of testing laboratories increased from 1 (Pasteur Institute) in March 2020 to 18, with PCR testing capacity totalling 5,000 per day in all 14 regions across the country by December 2020. GeneXpert equipment was set up in certain regions to increase capacity. The distribution of the testing laboratories was guided by the existing health system structure, which ensured the equitable distribution of services. Senegal is also manufacturing antigen RDTs locally.

The decentralized laboratories were supported by the government. At the beginning of the pandemic, only two laboratories were authorized by the State to conduct testing, and both were in the capital. Later, the government extended diagnostic testing to the regional level. It was necessary to wait for a secure technical platform before launching the diagnostic capacity in other areas.

The tests and inputs needed to use the devices were purchased by the government and routed to the decentralized laboratories. The laboratories were equipped with specific consumable equipment for COVID-19 (recalibrated to support COVID-19 diagnostics). The staff in some laboratories received support from, for example, Pasteur Institute staff who were deployed at the beginning of the pandemic (Darou Tanzil case in Touba) to help set up the laboratories and familiarize the personnel with the equipment. These staff members contributed to building the capabilities of laboratory workers, including the use of Genexpert.

*“In fact, we all used PCR*. *We have the GeneXpert in the lab and also the ministry often used them for the tests of tuberculosis*. *Now that, the Ministry of Health had received GeneXpert cartridges, the strategy was to distribute them to decentralized laboratories so that they be available at the level of the laboratories on the time of transmission and they could also be used for the urgent cases”* (*KI-1, Institut Pasteur, Dakar, Senegal*).

*“I think that the government has given support, at least in relation to the tests, especially support in reagents”* (*KI-1, Institut Pasteur, Dakar, Senegal*).

The COVID-19 outbreak was projected to be widespread in Uganda; thus, the National COVID-19 Task Force recommended different testing strategies and thresholds for adjustments, including the following: (i) if testing reached 500 samples daily at the Uganda Virus Research Institute (UVRI) laboratory, the country would activate mobile laboratories; (ii) if samples increased to 1,000 per day, other labs would be activated, e.g., the Joint Clinical Research Center; (iii) at 3,000 plus tests per day, the third level with higher output would be activated, including PCR equipment at the Central Public Health Laboratories (CPHL), known as Cobas 8800 (normally used for viral load monitoring for HIV); (iv) at >5,000 tests per day, private sector labs would be activated. This strategy was not strictly followed. Thus, the mobile labs were activated later than planned because they required imported point-of-care equipment. Additionally, there were procurement challenges as a result of increased global demand. The private sector laboratories were activated earlier than planned, likely because they had preexisting capacity. Testing capacity was expanded from 2,500 tests per day to over 8,800 tests per day as the number of laboratories increased from one in March 2020 to 16 in December 2020.^10^ The setting up of new testing sites or new labs was guided by the existence of infrastructure and the disease epidemiology (prioritizing high-risk POE and high-burden districts). By March 2021, the number of labs had increased to 21 because of continued certification.

Strategies implemented to generate testing capacity included leveraging existing laboratory capacity for endemic disease systems to support centralized PCR testing. The two major laboratories providing 90% of the PCR testing services, Uganda Virus Research Institute (UVRI) and the Central Public Health Laboratories (CPHL), were already supporting disease surveillance; UVRI provided HIV care services, and CPHL performed centralized viral load (VL) and Early Infant Diagnosis (EID) testing. This multiple disease pathogen testing, or “multiplexing” strategy contributed substantially to the rapid ramp-up of testing.

### 3.4. Improving access to testing

In the DRC, access to testing improved largely due to decentralization, although there were still major gaps in geographical coverage. Of note, 14 of the 26 provinces did not have a testing site as of December 2020. Samples were shipped by air to laboratories in the capital, Kinshasa, with the support of the WHO and CDC. Testing was provided free of charge to everyone except travelers. The introduction of POC RDTs improved access to testing. The introduction of payment for testing travelers was considered a major barrier, and there were reports that this approach may have reduced testing access. The “mass testing” implemented in Kinshasa (June 2020) increased access to testing. Testing access remained limited in the DRC, as only 6% of health facilities provided testing, and many provinces did not have a PCR testing site.

*“We have improved access to tests by decentralizing and extending the range of tests*. *Before, tests were only available at the INRB, then only at the provincial level, either the provincial laboratory or the laboratory that had the GeneXpert*. *Today with the antigenic tests, we have the HGR and the CS of reference which have tests and tomorrow we want the COVID tests to be like the RDT of malaria, that they are even available to the community because the ideal is to test everyone”* (*KI-3*, *Program National de Lutte contre la Tuberculose, Kinshasa, DRC*).

*“At the beginning, there was a big delay in the delivery of results, but then there was an improvement because the private sector took over; in terms of change, it was significant during the response; the introduction of Genexpert equipment lightened the load and allowed some provinces to do the tests on site*.*”*

*“Per capita testing has not improved at the state level, but the private sector has taken over*. *The country does not have the capacity to produce test kits locally”* (*KI-8, Director of the Health Laboratories Division, Kinshasa, DRC*).

In Nigeria, in May 2020, the existing (10) national GeneXpert equipment previously used for TB diagnostics was recalibrated to make it available for COVID-19 testing.^11^ The addition of GeneXpert testing allowed for a major increase in testing numbers and improved the TAT. However, the lack of skilled human resources persisted even with this strategy. In addition, TB diagnostics at one center decreased by 30%.^12^

In September 2020, antigen RDTs were incorporated into the testing algorithm. Two RDTs (Abbot and Biosensor) were approved for use in special settings and are currently being piloted in the National Youth Service Corp (NYSC) camps. RDTs have not been recommended for replacing PCR testing due to sensitivity and specificity limitations. The current recommendations for the use of the Ag RDT test are in the following contexts in Nigeria:

Health care settings: Testing of health workers for COVID-19 and patients with symptoms of COVID-19 presenting in hospital triage areas. A positive RDT test confirms SARS Cov-2 infection.
Contacts of PCR confirmed cases: A contact who tests positive using AgRDT is considered confirmed positive for COVID-19. If the AgRDT is negative, the person is considered negative.
Closed settings: Boarding houses, prison inmates, NYSCs, and other similar closed settings.

The first case in each setting that is positive on an AgRDT should be retested by PCR for confirmation. Once a positive test has been confirmed by PCR, all subsequent AgRDT-positive results are considered confirmed and retesting is not required.

Access to testing has improved over time in Nigeria due to an increase in the number of sample collection and testing sites. Other strategies included the provision of free testing at public locations (except for travelers) and regular communication to the public regarding where they could access testing. All COVID-19 testing was provided for free except at the fee-paying labs shown in the map. One key issue observed was an inadequate demand for testing services, with underutilization of the established testing capacity. In response, the NCDC conducted a campaign to test for COVID-19 when people experienced influenza-like symptoms. Other key barriers to access the long distances people were required to travel to the few sample collection and testing sites, the long TAT for results, stigma and misconceptions in the community, and poor dissemination of information regarding testing center locations.

These barriers were addressed by providing health education to the communities on the need to address stigma as part of risk communication. The testing cost for travelers was also thought to be a limitation. Excessive demand for testing was noted among people in high positions who did not meet the testing criteria but demanded testing alongside their family members and staff. Nigeria has increased the number of COVID-19 testing laboratories from 3 to 97 (as of Jan 2021) since the beginning of the pandemic in 2020. These laboratories are spread across the 36 states and FCTs, with 79 laboratories open to the public at no cost to ensure that Nigerians can access testing when needed. However, international travelers who require a negative PCR test result before traveling must pay for testing at an accredited private laboratory of their choice. The public laboratories are intended for in-country response efforts and are not to be used by passengers coming in or out of the country. Overall, the laboratories (public and private) have the capacity to test at least 15,000–20,000 samples daily (January 2021).

*“So, for the testing strategy there were some public health laboratories across in some states even before COVID-19*. *We have this network of laboratories but with COVID-19 and the cases being reported in all the states and with the needs for us to have prompt diagnoses made, which will inform your next action, so there was a need to actually established more labs in more states, so these laboratories were established in many states*. *So, we have a number of states which never had laboratories before, which were sending their samples to other states before which at sometimes led to some delay in the testing but with the establishment of labs in these other states it improved the turnaround time for the samples and we also got support from partners, especially some machines that were being used for TBs and all that which are also used for this testing, so these are some of the testing strategies that were modified as the response activities went on”* (*KII-15 National EOC, from NCDC Abuja*).

*“What actually affected the turnaround time in the lab is dearth or insufficient reagent, that is the only, at least in the lab where I was seconded to, I would say that was the main thing that is obvious to me, not the personnel, not the facilities, there were ready 24 h, but the reagent, once they ran out of reagent before they sent it from Abuja to that side it takes a long time, so at times they now have batches they will now be releasing result in batches after some time but once they have it result would be coming out in good time so those were the major challenges*. *So the other one is they realized that coming to a center position will be faster than the time it would take for someone to commute from point A to point B wanting to get samples, you will save time if those people themselves come to a convergent place and then you are there to just take all the sample and then immediately go to the lab, so the number you would do would be better that’s why I actually applauded creating collection site, just to increase the number of samples been tested”* (*KI-10, National Laboratory of Oyo State, Nigeria*).

In Senegal, access to testing improved largely as a result of the decentralization of testing services and deployment of POC tests and RDTs. In addition, testing is provided free of charge, except for outgoing travelers, who pay the equivalent of US$70.

*“The fact, people can be tested in all health centers*. *The fact that Senegal already had a well-oiled surveillance system*. *The fact that they have set up mobile laboratories even in other regions*. *The fact that the tests in Thiès are done by the IRSF, we have decentralized the tests to the other regions”* (*KI3, Dialal Diam Hospital, Senegal*).

*“There are two things, for the patients there is no problem*. *The doctor who suspects that a person has COVID, asks for the test and in general there is no problem for this patient to have the test*. *Now the problem is with the travelers who had to take the test because they had to pay, go to the laboratory and wait in line to be tested*. *But for the patients who were in the suspect group, they got their test in time and it’s free”* (*KI-18, WHO, Senegal*).

Uganda improved access to testing through the decentralization of services and an increase in the number of sample collection and testing sites. The sample collection and testing sites were widely publicized in the media and through Ministry of Health (MOH) communications. Testing was provided free of charge to everyone except for travelers. The notification system in the community and measures introduced to pick up samples from those suspected of being infected improved access to testing. Prior to the COVID-19 pandemic, Uganda had a preexisting strong national health laboratory system with comprehensive infrastructure at the central and subnational levels. Many laboratories had the capacity to perform PCR testing. In addition, there was an established sample transport network linking 97% of national health facilities to testing labs.

Improving TAT: The turnaround time was reduced from 2 to 3 weeks in the early phase of the epidemic (March 2020) to 24–48 h for provinces with a testing site (July 2020) in the DRC. This reduction was largely due to the decentralization of testing services, i.e., the long TAT for results led the MOH to change strategy and decentralize testing services. However, provinces without a testing site still have their samples routed to a laboratory through Kinshasa by air, with a median TAT of 7 days. The long TAT was a major challenge to surveillance, as there were delays in decision-making, including the isolation of individuals with confirmed cases and contact tracing. The laboratory results were returned electronically through phone calls, SMS texts, or emails before a hard copy was shared with the health professional or the individual tested, e.g., a traveler.

In Nigeria, the TAT from sample collection to result return was 24–48 h for PCR testing and under 24 h for GeneXpert testing as of November 2020. This is a significant improvement from 5 days in the early phase of the outbreak. Improvement has been realized as a result of increased laboratory testing capacities, the decentralization of services, adoption of POC testing and RDTs, and improvements in the sample transportation system. Dedicated sample collection teams were coordinated and trained to support sample transport. The key challenges noted were the poor road network, logistical complications, and weak coordination capabilities across the diagnostic testing laboratories. For example, there were reports of sample delivery to laboratories that were not properly labeled and could not be directly linked to a particular source, and such samples were destroyed. This challenge was precipitated by the inadequate supply of testing kits and PPE, poor synchronization between surveillance and laboratory data and long TATs. The results from public and private laboratories are returned to the NCDC and state government EOC, from which they are communicated to the tested individuals and health providers. The results return leverages a preexisting system within a database at the office of the Disease Surveillance and Notification Officer at the local government level, which routinely collects and collates facility-level data into the District Health Information System (DHIS2) for real-time transmission and collation at the state and federal levels. Following the COVID-19 outbreak, the testing labs were linked to the states’ DHIS2 IT infrastructure to enhance the data transmission speed.

Although the TAT has improved, further improvement is required, as evidenced by the comments of the key informants below:

*“TAT is still a challenge*. *Tests that should be within 2/3 days are delayed till 2 weeks and by the time you have test results coming after 2 weeks, the person is no longer infectious even if the person was positive”* (*KII-4, Surveillance Pillar Member, State EOC, SS*).

*“The turnaround time of result makes it difficult at times*. *In some cases, the incubation period would have elapsed before the result is out*. *It affects surveillance because the confirmed cases that have exceeded the incubation period are not ready to cooperate with us most of the time”* (*KII-12, Laboratory Team Member, State EOC, SW*).

In Senegal, the TAT from specimen collection to results return improved from 2 to 3 days to 9 h over 3 months (March, April and May 2020). Strategies that contributed to the improved TAT included the decentralization of testing services, the use of POC testing (including RDTs), and the expedition of results return. The results were communicated through phone calls and in writing via the internet, for example, email notifications.

In Uganda, the TAT improved by 12–72 h on average between March and October 2020. The TAT averaged 2–7 days in March 2020 and decreased to 8–24 h in October 2020, although the range varied widely depending on the laboratory and geographical location. Districts furthest from the central labs reported TATs as long as 2 weeks. The TAT was the shortest in areas with POC testing. However, given that most of the testing was centralized, efforts were made to improve efficiencies in the transport network, including a notification system that alerts the central laboratory and the district that a sample has been collected. Other strategies to improve the TAT included the use of a sample tracker system, an increase in the number of vehicles for sample transport, the automation of laboratory systems where possible, and the use of electronic systems for results transmission from the laboratory to the EOC, district and facility. The adoption of RDTs began in December 2020 to further reduce the average TAT.

Additional work shifts were introduced and volunteer staff were deployed to further increase testing capacity in these laboratories and reduce the TAT. Some laboratory capacity/equipment was reserved for VL, EID, surveillance, and other disease support at both CPHL and UVRI to minimize the risk of the displacement of the diagnosis of other diseases. A further increase in capacity was realized through (ii) partnerships with the private sector (including academia, private for-profits, and private non-profits). This strategy increased testing access and reduced the burden of testing on the government. This strategy also provided a suitable option for individuals who wished to test but were not eligible for free testing, such as travelers. (iii) New mobile laboratories were established at three border POEs to test truckers along trade routes since the majority of the early COVID-19 confirmed cases were imported through these POEs.^13^ These mobile laboratories utilized GeneXpert equipment and improved efficiency and the TAT at the borders from 2 to 7 days to 12 h. The establishment of mobile laboratories at the border strengthened disease surveillance. (iv) Pooling of samples was implemented at a few laboratories, including CPHL and Makerere University.

*“The capacity has improved so much*. *So the testing capacity has gone very high especially with the introduction of RDTs*. *Then, increase in the number of laboratories especially private laboratories and academia from universities has also increased this*. *The introduction of other testing platforms like RDTs and gene extract have also increased so the testing capacity has gone very high although the reporting is known to be poor*. *So that is the general comment I can give about the testing capacities for COVID 19 across the country*. *But it needs an enhanced quality check on the old system*. *There are a number of quality issues as the capacities are being expanded so rapidly there these issues across the country*. *There is a need for enhanced supervision and that type of thing by CPHL or the department of the laboratories”* (*KI2, CPHL, MoH, Uganda*).

Furthermore, a laboratory information management system with an electronic results transmission and download system was also introduced. This system helped to improve the TAT. The established laboratory network was leveraged to improve COVID-19 testing access. However, one of the challenges encountered was that people still wanted to be tested at UVRI. To mitigate this, UVRI reassured the public about the quality of the testing offered by the accredited laboratories.

*“For PCR, we have over 25 labs; they must be close to 30 by now because yesterday I got to know that there are two under the pipeline for activation and some of these labs are private for profit*. *That has been the game changer and it is something that I thought I should mention as a new improvement after the pandemic for the last 3 months*. *First of all, the ministry guided on the testing; I’m sure the first pricing which was at 60USD that they mentioned some time back has been dropping and many labs are testing below 60USD*. *It is still high but there is even more hope for prices dropping and so a large number of populations are able to access timely results when they need it*. *In total maybe I can say the capacity we usually have for public labs comes from CPHL where they can produce about 4,000 tests per day and UVRI which can produce about 2,000 tests per day though their average is 1,000 or 500 tests per day*. *The rest of the other labs are becoming commercial; they are reserving it for commercial purposes*. *I should mention Makerere which is using a cost recovery means and their tests are way cheaper than compared to any other lab*. *You can have a test at maybe Shs*. *180,000 and about 30USD; something of that sort*. *Makerere can do quite a lot like about 4,000 tests per day; like I said, I can get you that capacity that is well calculated and we have it on paper”* (*KI1, CDC, Uganda*).

The four countries had in existence public and private laboratory testing platforms previously supporting clinical diagnostics, surveillance, and research for TB (using GeneXpert), HIV, influenza, Ebola, Lassa, and others. These systems were leveraged to support COVID-19 testing. Initially, at the onset of the pandemic, the biggest issue was lack of funding for procurement of testing reagents, supplies and PPE. However, even when funding was made available through donations from the various partners and government. The countries decentralized COVID-19 testing services by leveraging preexisting laboratory systems such as polymerase chain reaction (PCR) and GeneXpert used for the diagnosis of tuberculosis (TB) to address this gap and the related inequities, engaging the private sector, establishing new laboratories, and using rapid diagnostic tests (RDTs) to expand testing capacity and reduce the turnaround time (TAT). Access to testing improved largely due to decentralization, mass testing (free testing), and increase in the number of sample collection and testing sites.

## 4. Discussion

This paper describes experiences and lessons learned while improving testing capacities during the COVID-19 response in the DRC, Nigeria, Senegal, and Uganda, informs on the response to COVID-19 and provides information for addressing future pandemics in these regions and worldwide.

As of January 2, 2021, Senegal and Uganda had performed more tests *per capita* than the DRC and Nigeria. However, collectively, the testing rate was low in all the countries. The low testing output *per capita* or per million population was related to (i) inadequate testing capacities (a limited number of trained staff or lack of equipment); (ii) limited access to testing sites (poor road and several provinces without a PCR testing lab); (iii) inadequate testing supplies (due to challenges in sourcing supplies from global manufacturers; inadequate funding); and (iv) implementation of a testing strategy that targets only high-risk individuals. Seidu and colleagues have generally explained the reasons for the low testing in African countries. These countries require an intense upgrading of their testing capacities because of their limited capabilities in testing centers and existing diagnostic facilities with inadequate personnel and reagents (11). To make the most of scarce testing resources and to reach individuals in urgent need, one approach may be to target symptomatic cases only (12). With this high-risk strategy, some infected people went unnoticed because of testing-related challenges, including persons with COVID-19-compatible clinical disease (11, 13). This testing strategy has also been implemented in the DRC and Nigeria because of their low testing capacity. The low testing outputs in DRC and Nigeria could also be explained by their high populations compared to those of Senegal and Uganda, of 85 and 206 million vs. 17 and 42 million, respectively. As of June 19, 2020, Nigeria, the most populous African country with a population of over 206 million people, was only able to test 106,006 people across its 30 testing sites, illustrating the lack of laboratory testing capacities (14, 15). Notably, Senegal manufactured COVID-19 testing kits at a low price, thus facilitating access to testing (16), which could justify the relatively low test *per capita* ratio in the DRC and Nigeria. Universal access to diagnostic testing is crucial to control an epidemic; hence, mass testing strategies remain the international best practice (12).

### 4.1. Partnerships

At the onset of the outbreak, testing supplies (including PPE) were primarily sourced through donations or procured with funds from international partners, including the European Union and World Bank.

Dzinamarira et al. (17) explain that the challenge for most African countries went beyond the poor health system and arose from their economies. However, the countries that could provide assistance were themselves battling to control the pandemic for their own populations (17). Africa is not new to epidemics (18). Repetitive epidemics have left countries with some relevant infrastructure, such as diagnostic testing and surveillance systems. Thus, as shown in this study, after the initial minimal responses, when the reported number of cases reduced, aggressive responses were announced in many countries (17, 19). Partnership with donors, the private sector and funding agencies remains highly recommendable (19), as we have also indicated.

According to del Rio and Malani (20), developing vigorous testing capacities is necessary to address the current outbreak, verify persons with the virus, and identify those who are asymptomatic. Therefore, each African country must establish COVID-19 testing capacities, perhaps first collaborating regionally. Countries with minimal testing capacities could be encouraged to refer samples from suspected cases to a WHO reference laboratory for COVID-19 testing through interlaboratory collaboration. Countries with developed testing at the national level could scale it up by decentralizing testing through identified regional laboratories. Additionally, private and/or academic institution laboratories meeting the required standards could be included, especially in geographical locations with limited facilities (4, 11). Public–private partnerships for COVID-19 screening have been encouraged and practiced in most African countries due to insufficient diagnostic tests at government-owned facilities.

### 4.2. Lack of testing supplies

Inadequate testing supplies limited the increase in testing capacity and access to testing, with countries establishing suboptimal numbers of sample collection and testing sites. Initially, at the onset of the pandemic, the biggest issue was a lack of funds for the procurement of testing reagents, supplies and PPE. However, even when funding was made available, procurement was hampered by inadequate production at the global level and delayed delivery of commodities as a result of COVID-19-related travel restrictions. Within countries, there was rationing of commodities with reported stock-outs in all countries, except Senegal. The test requires expensive equipment and highly trained personnel; hence, in most African countries, very few centers can run COVID-19 tests. Most of these tests are no longer manufactured in Europe or America, and very few African laboratories are capable of manufacturing them; only 1–3 laboratories among the 40 present in the region have this capability (21). The lack of tests in most African countries increased the turnaround time of the results. Hence, at one point, the strategy was to propose tests that would give results within 30 min following the collection of a sample (21). Kobia and Gitaka (21) found that in many contexts, obtaining results takes days (21). Consequently, laboratory testing of suspected cases is characterized by long waiting periods and an exponential increase in the demand for tests. The turnaround time is another significant limiting factor in PCR testing, given the duration before the diagnosis is issued.

### 4.3. Limited testing capacity at onset

Several strategies were proposed to address the difficulty of screening at the beginning of the pandemic, especially decentralization, which means allowing some peripheral laboratories to also organize screening; RDTs were also proposed. At the beginning of the pandemic, the turn-around time was very high, which obscured the extent of the outbreak in the different countries (11). At the beginning of the pandemic, high-income countries were heavily hit with a high number of deaths; thus, all the equipment related to diagnosis was still concentrated in these countries, thus making testing difficult in African countries.

### 4.4. Long TAT for PCR tests (3–7 days)

At sites where point of care (POC) testing approaches had been adopted, including GeneXpert platforms and RDTs, the TAT had been reduced to minutes or hours. The limited capacity to screen for COVID-19 in African countries was due to several factors, including the use of PCR. PCR was, however, the test that offered highly precise and sometimes quantitative SARS-CoV-2 RNA detection. This test is slow to execute and costly. The RT–PCR test kit can cost more than 100 US dollars, and it takes more than 15,000 US dollars to set up a diagnostic laboratory (14, 22). Of note, the analysis time of RT–PCR in most African countries is not less than 4 h, and the turn-around period is more than 24 h, from sample collection to the readiness of the result (14). Due to the lengthy process of using RT–PCR, the use of other screening techniques has been implemented in sub-Saharan African countries, including the use of RDT, which, unlike RT–PCR, does not require the extensive training of personnel, and the result is delivered in a short time (10). African countries with a high prevalence of tuberculosis have used GeneXpert to diagnose COVID-19 and improve the screening system in general (21).

### 4.5. Stigma and widespread misinformation

Stigma, myths, and misinformation contributed to the low testing demand in some communities. This observation was reported in all four countries, especially Senegal and Nigeria. In South Africa, evidence from past outbreaks, such as HIV, has also shown that stigma could prevent people from seeking treatment (23). This is also true for COVID-19 (24). The groups of people at high risk for stigmatization are truck drivers, sex workers, migrants, returnees, and displaced individuals (25, 26). Efforts to control the cross-border transmission of COVID-19 can be hampered by political and economic factors that can cause people working in the cross-border area to be unwilling to undergo testing (27). However, worldwide, the pandemic has impacted the mental health of millions of people by increasing levels of fear, stress, anxiety, and uncertainty. This emotional instability has also resulted in attacks on health care personnel for fear that they will transmit the virus to those around them (28).

### 4.6. Inadequate human resources for testing

All countries reported inadequate staff with skills for molecular testing. However, this was mitigated through rapid training. Many African countries have diagnostic saturation since conducting a daily target number of detection tests, especially viral RNA, by RT–qPCR requires great organizational and logistical rigor. As a result, the shortage of qualified personnel was evident. African regions performed approximately 20–70 times fewer diagnostic tests *per capita* than the three most affluent western regions (North America, Northern Europe, and Mediterranean Europe) (29). In Burkina-Faso, human resources were insufficient, and people were poorly qualified and poorly motivated to respond to COVID-19. The COVID-19 pandemic revealed the shortcomings of their health system in terms of controlling the outbreak, as it does not correctly reflect the reality of the outbreak. Only cases requiring treatment in the hospital were tested (30). This lack of qualified personnel contributed to the delay in the implementation of the various recommendations and guidelines developed for the control of the pandemic in Africa.

### 4.7. Existence of in-country laboratory capacity for diagnosis of endemic diseases

The WHO-recommended COVID-19 testing modality at the onset of the pandemic was the PCR test. The four countries had in existence public and private testing platforms previously supporting clinical diagnostics, surveillance, and research for TB (using GeneXpert), HIV, influenza, Ebola and Lassa fever. These systems were leveraged to support COVID-19 testing. Other member states of the African Union were able to achieve their diagnostic capacities at the subnational level by fingerprinting PCR test platforms used in other national disease control programs.

Ethiopia increased its capacity to 7,600 tests per day after Abbott agreed to reconfigure its closed platform to accommodate COVID-19 tests. This act by the Abbott laboratory was followed by universities and animal health laboratories (3). However, this practice of reorienting materials initially allocated for other infectious diseases had a negative impact on the diagnosis of HIV and tuberculosis. The supply of reagents was also a major challenge for the continuity of services. Some West African countries, such as Niger and Guinea, increased the diagnostic capacity of their laboratories following many Ebola epidemics that their countries experienced (31).

The different rapid tests used in the four countries were important in providing speedy results and thus increasing the diagnostic capacity and curbing the spread of COVID-19 (14). The antigenic test allows the detection of an active infection even among nonsymptomatic people, although it is also limited by the viral load, the quality of the sample taken and, especially, the duration of the infection.

### 4.8. Local development and manufacturing of RDTs

Senegal began local manufacturing of antigenic RDTs in July 2020 through a private partnership with Diatropix. Activities were officially launched in November 2020 and started slowly. However, in August 2021, as Senegal was experiencing the third wave of COVID-19, the Pasteur Institute in Dakar gave 50,000 test kits to the government to quickly stem the epidemic (32). The objective was to produce 200,000 tests per month from the start of 2022 and eventually 2.5 million tests per month according to the requests of the countries of the continent (33).

### 4.9. Achievements and challenges with COVID-19 testing

As achievements, all the four countries leverage existing capacity, adopt RDTs, use of digital platforms, and create a partnership for sourcing and capacity.

### 4.10. Challenges

Insufficient detection of cases: Test positivity rates were often high during the pandemic, indicating that many cases went undetected. This was validated by serology studies, Serological survey in Nigeria found much higher incidence than reported.
Inadequate access: Major gaps in testing access have persisted, with some facilities unable to offer tests or pricing tests beyond what is affordable.
Sourcing of limited supplies: Supplies for test kits, PPE, and sample collection were inconsistent and supply chains were strained by the increase in demand. Even when funding was available, countries faced difficulties competing in the global market, DRC relied on unapproved RDTs given lack of PCR test supplies.
Ensuring quality: Testing often performed across many labs with gaps in quality; RDTs still not accurate enough for use beyond screening/triage of symptomatic, Reports in Uganda of different results from different labs.
Creating demand: Misinformation on testing created stigma, and access to facilities hampered demand.
Turnaround time: Most countries faced challenges getting turnaround time within a 24–48-h window.

Challenges mitigation may include the following key strategic actions: (1) roll out of approved RDTs for COVID-19 testing; (2) validation of new RDTs as they become available; (3) support for local production of RDTs; and (4) further decentralizing PCR testing. Expanding PCR testing capacity will not only serve to increase access to reference labs providing confirmatory testing for COVID-19, but the labs could potentially serve as surge capacity for future disease outbreaks through multiple disease pathogen testing.

### 4.11. African countries may adapt a model of integrated respiratory lab network

There were many challenges related to the management of the supply chain which affected availability of testing supplies. Due to the rapid spread of the virus and huge volume of supplies for infection prevention and control (IPC), there is more than ever an urgent need to have strong supply chain mechanisms. Pooled procurement mechanism (PPM) worked well for Global Fund procurements. The PPM aimed to provide access to competitive market terms and prices, no matter the order’s size or value; eliminate procurement delays due to complicated tendering processes, support timely grant expenditure; and ensure that quality assured goods and medicines reach those most in need in a timely manner.^14^ The PPM could be consolidated at regional level to support future pandemics, meaning to set up regionally coordinated pooled procurement and manufacturing mechanisms.

## 5. Limitations

The results and interpretations of our findings are subject to several limitations especially that we conducted the work in the middle of a pandemic, which might have limited access to key informant interviews with some important policy-makers and access to government documents. However, the findings are important as countries continue to respond to the COVID-19 pandemic and prepare for other epidemics in the future.

## 6. Conclusion

While testing is essential for disease detection and surveillance, access to COVID-19 testing was still limited across the four countries assessed in this study due to inadequate resources to meet the demand for PCR/molecular testing, including equipment, skilled personnel, reagents and supplies.

The sample collection and testing sites remain limited, and the turnaround time for test results is too long, especially due to the need to transport samples from collection sites to testing labs. The approved RDTs are not yet widely available, and their utility is still limited due to their low sensitivity and specificity.

However, testing capacities can be improved through the wider adoption of simplified testing approaches, including the use of RDTs as part of comprehensive testing algorithms and the further decentralization of PCR testing capacity.

Further investments are needed for countries to develop local capacities for the production of supplies for COVID-19 testing, including the development of new and more accurate RDTs.

The key findings are summarized in Table 1.

**Table 1:** Summary of key findings.

Components	Cross-country actions
Partnerships for sourcing and capacity	Donations and funding from partners were critical for increasing testing supply and procurement. Additionally, coordination with private labs was essential for scaling test capacities and manufacturing.
Expanding testing capacity	Testing services were decentralized. The expansion of COVID-19 testing sites was guided by the availability of infrastructure (human resources and GeneXpert equipment); disease epidemiology, with more laboratories in Kinshasa (the epicenter) and considerations of geographical equity.
Leverage existing capacity	Endemic disease systems and existing transport capacities were significantly ramped up (Use of Gene Xpert for TB in DRC, Senegal, and Nigeria).
Improving the TAT	Improvement has been realized as a result of increased laboratory testing capacities, the decentralization of services, the adoption of POC testing and RDTs, and improvements in the sample transportation system.
Key learnings	Set up regionally coordinated pooled procurement and manufacturing mechanisms.
	Risk-based testing strategy (e.g., prioritizing the older adult or travelers) limits the comprehensive understanding of the disease burden.
	Simplify testing modalities when possible.

## Data availability statement

The raw data supporting the conclusions of this article will be made available by the authors, without undue reservation.

## Ethics statement

The studies involving humans were approved by Kinshasa School of Public Health. The studies were conducted in accordance with the local legislation and institutional requirements. The participants provided their written informed consent to participate in this study. Written informed consent was obtained from the individual(s) for the publication of any potentially identifiable images or data included in this article.

## Author contributions

MB, RW, AN, SNKi, SNKa, MM, OF, and IS conceptualized the study. MB, LE, YK, DK, BB, and MM conducted the data collection, analysis, interpretation, and review of country findings from DRC. OF, SB, EB, MD, AA, MS, and RA conducted the data collection, analysis, interpretation, and review of country findings from Nigeria. IS, ID, ML, MF, and OB conducted the data collection, analysis, interpretation, and review of country findings from Senegal. SNKa, SNKi, RN, SKiz, FM, NN, AN, and RW conducted data collection, analysis and interpretation, and review of country findings from Uganda. AN, SNKa, SKiz, RN, and RW led the cross-country synthesis. MB wrote the first draft of the manuscript. All authors contributed to the article and approved the submitted version.

## Figures and Tables

**Table:** 

CHAI	Clinton health access initiative
COVID-19	Coronavirus Disease 2019
CPHL	Central Public Health Laboratories
DHIS-2	District Health Information System
DRC	Democratic Republic of Congo
EOC	Emergency operation center
EID	Early infant diagnosis
GHSI	Global health security index
HIV	Human immunodeficiency virus
INRB	National Biomedical Research Institute (DRC)
JICA	Japan International Cooperation Agency
LMIC	Low-and Middle-Income Country
MOH	Ministry of Health
MSF	Médecin Sans Frontière
NCDC	Nigerian Center for Disease Control and Prevention
NYSC	National Youth Service Corp
PCR	Polymerase chain reaction
PNLT	National Program for Tuberculosis control (DRC)
POC	Point of care
POE	Point of entry
RDT	Rapid diagnostic test
RNA	Ribonucleic acid
TAT	Turnaround time
TB	Tuberculosis
UNICEF	United Nations International Children’s Emergency Fund
USAID	United States Agency for International Development
UVRI	Uganda Virus Research Institute
VL	Viral load
WHO	World Health Organization

## Footnotes

^1^Global COVID-19 Tracker—Updated as of August 26: https://www.kff.org/coronavirus-covid-19/issue-brief/global-covid-19-tracker/

^2^Our World in Data. Senegal: Coronavirus Pandemic Country Profile. Available at: https://ourworldindata.org/coronavirus/country/senegal

^3^ https://www.worldbank.org/en/news/press-release/2020/04/02/the-world-bank-group-provides-47-million-to-support-the-democratic-republic-of-congos-response-to-the-coronavirus-pandemic

^4^ http://www.pasteur.sn/inauguration-de-la-plateforme-de-production-de-tests-de-diagnostic-rapide-a-linstitut-pasteur-de-dakar/

^5^ https://www.fondation-merieux.org/actualites/inauguration-de-la-plateforme-de-production-diatropix-a-linstitut-pasteur-de-dakar/

^6^ https://www.lemonde.fr/afrique/article/2020/04/13/dakar-va-produire-des-tests-rapides-de-depistage-du-coronavirus-a-moins-d-un-euro_6036469_3212.html

^7^ https://dochub.com/creativethinkt3/JWop0ZAKk2x4WXNRrYa9GP/covid19-list-of-donations-in-uganda-pdf?dt=os7xiPFxZnKqzv5RHPUd

^8^Homegrown COVID-19 Rapid Antibody Test kits Launched by Makerere University - East African Business Week. https://www.busiweek.com/homegrown-covid-19-rapid-antibody-test-kits-launched-by-makerere-university/

^9^ https://covid19.ncdc.gov.ng/laboratory/

^10^Uganda Ministry of Health. Accredited laboratories to carry out COVID-19 testing in Uganda. 2020.

^11^Existing Genexpert laboratories recalibrated for COVID-19 were 23 (https://covid19.ncdc.gov.ng/laboratory/).

^12^ https://theunion.org/news/impact-of-covid-19-on-tb-care-experiences-of-a-treatment-centre-in-nigeria

^13^ https://ugandaradionetwork.net/story/covid-19-mobile-lab-finally-launched

^14^Audit report: Procurement and Supply Chain during the COVID-19 pandemic.
